# Supplementary material for: Airway management during ongoing chest compressions–direct vs. video laryngoscopy. A randomised manikin study
Source: PLoS One. 2023 Feb 9;18(2):e0281186. doi: 10.1371/journal.pone.0281186 (PMC9910718; doi:10.1371/journal.pone.0281186)
Supplement: Attachment — Submitted filename: Revision PLOS ONE_clean.docx [file pone.0281186.s002.docx]

| **Journal Requirements** |  |
| --- | --- |
| The discussion has not highlighted the important limitation of the small sample size and lack of power calculations associated with the study. | We have now described these limitations of the study in more detail in the discussion. |
| There is limited discussion with regard to the multivariable logistic regression modeling used in the study. | Thank you for this hint. We have interpreted the results of the regression analysis in more detail in the discussion section. |
| An important limitation of the model is that other potential confounders were not included to fit the model as these were not measured but could have been measured. This is worth highlighting in the discussion (e.g Age, experience with intubation-even though inferred by the variable "experience in anesthesiology"). | Age is not correlated to experience in airway management at all. A 30 year old doctor starting his career in anaesthesia at an age of 28 for sure has less experience in airway management as a nurse at an age of 30 who started his/her career at an age of 22.  We did not include the experience in tracheal intubation as a variable in our regression model for two reasons: it would have been only a rough estimate by the participants and it would closely correlate with the working experience in anaesthesiology in our clinic. We mentioned this limitation in the corresponding section: “Fifthly, we did not include the experience with tracheal intubation as potential confounder for our multivariable regression model. However, this variable would very closely correlate with working experience in anaesthesia in our clinic at a university hospital. Therefore, we constrained to the working experience in anaesthesia as a simply and objectively measurable parameter.“ |
| Although goodness of fit assessment was done and the result presented with Table 4, there is no discussion of these results to assist readers in understanding how well the data fit the model. | We performed a multivariable regression analysis in order to test whether working experience in anaesthesia or type of profession is a predictor for first pass success in tracheal intubation. Both variable have been proven to be *no* predictors (working experience in years: OR 0.98 [0.91-1.06] and type of profession nurse OR 0.51 [0.09-2.55]). So “goodness of fit” of this model (with two variables that are no predictors) is expected to be poor.  We added our parameters on “goodness of fit” to Table 4 as this is standard in presenting results of regression analyses. However, these parameters have only little (if any) relevance for the interpretation of our results in this case. For this reason, we did not discuss these results in our manuscript.  We would like to let the editor make the decision, whether we should explain the poor “goodness of fit” to the readers in our discussion. However, this paragraph would be little bit out of the scope of this article. |
| **Reviewer #1** |  |
| 1. Authors state tracheal intubation is the gold standard during CPR. This is not true. Bag mask ventilation has the same weight/importance. | Thank you for this. Your statement is of course absolutely correct, we have adapted the corresponding statement in the introduction. |
| 2. The FPS was higher in the VL group and the TTI was shorter. However, the difference in TTI was only 2.5 seconds. This seems to be clinically insignificant, especially during CPR (at least in non-hypoxic causes). Moreover, if the 9 unsuccessful first intubation attempts were also counted in the TTI it further diminishes importance of these findings. | We entirely agree with you that this difference in TTI may not be clinically relevant. We therefore deliberately chose the FPS as the primary endpoint. The inclusion of the 9 unsuccessful intubations would increase the difference in the TTI significantly, but this does not correspond to clinical practice: in the case of unsuccessful intubation, interim ventilation (using bag-mask ventilation) should be given promptly and the next intubation attempt should be made after optimizing the conditions (e.g. positioning the patient, suction) or with an alternative airway device (e.g. SGA). We feel, it would therefore be ill-advised to include these intermediate steps in the pure TTI. |
| 3. As stated by the authors, crossover design would be much more appropriate. | We agree that a cross-over design could potentially offer benefits. However, for logistical reasons, it was not possible to summon all participants again with a sufficient time difference. In this setting, the two intubations would have taken place within a short period of time and we then would expect a significant bias due to the short-term experience and training effect. This would not meet our claim of a pragmatic trial simulating the setting of prehospital emergency care medicine. We discussed this limitation in more detail in the corresponding section. |
| 4. Do I understand it right, that a VL with external monitor was used? In this setting (ongoing CPR) a “screen on the handle” device would be more appropriate (transport reasons). From a clinical practice, it seems to me, that using the “screen on the handle” device might be more difficult than using the external monitor. | That is correct, we used a VL with an external monitor for this study. We agree with you that in preclinical practice, a device with a screen “on the handle” is usually used. We also agree with you that this could make intubation more difficult. However, we considered it more important for our research question that we were able to create conditions that were as standardized as possible for both groups. Thus, both groups had exactly the same handle, the only difference was the view of the monitor. We have also now described this accordingly in the text. |
| 5. Did all of the participants intubate manikin before? Or were there differences? | We intentionally used the same mannikin for our experiment, which is repeatedly used in simulations and courses at our clinic. All participants have completed at least one training session with this equipment (all employees undergo standardized basic life support training when starting their job). However, it must be noted that intubation of the manikin is not performed as part of this training. Thus, the experience levels in this regard is without differences. |
| 6. Statement in the discussion that VL should be recommended for tracheal intubation during CPR is too strong and not supported. | We have re-worded the relevant passage. |
| 7. A chapter alone are SGA devices. For an inexperienced rescuer, the use of SGA might be easier, faster and also without chest compression interruptions. | We clearly agree with you on this point. However, we believe that the evidence on choice of airway management in OHCA is still insufficient. In older, large randomized studies, no clear statement could be made on the superiority of one or the other modality in relation to a favourable neurological outcome after 30 days (e.g. AIRWAYS-2). However, for example, Benoit et al (DOI: 10.1016/j.resuscitation.2020.04.015) recently showed a possibly better outcome in intubated patients and tended to prefer endotracheal intubation in this situation - especially in well-trained physicians. The PART study by Wang et al. concluded that an initial laryngeal tube placement strategy was associated with a significantly higher 72-h survival rate than an initial tracheal intubation strategy. However, this study has the major disadvantage that the FPS in patients who were tracheal intubated was only 51%. Overall, the trend in the recent literature seems to be that tracheal intubation may be associated with higher short- and long-term outcome rates and better neurological recovery (e.g. Behrens et al DOI: [10.1016/j.resuscitation.2020.04.015](https://doi.org/10.1016/j.resuscitation.2020.04.015) or Sulzgruber et al. DOI: [10.1177/2048872617731894](https://doi.org/10.1177/2048872617731894) ).  Nevertheless, the possible benefit of tracheal intubation (if used by experienced providers) should not be diminished by no-flow-times for laryngoscopy and tube placement. Or formulated differently: the benefit of SGA as shown in some studies might be due to shorter no-flow-times compared to tracheal intubation. Possibly, the best strategy is placing a tracheal tube using a VL with no interrupting of chest compressions? Our study provides the first step to explore this hypothesis.  We discussed this issue in more detail in our manuscript. |
| **Reviewer #2** |  |
| The suggested keywords are all appropriate (although airway management is spelt wrongly). Others could be added e.g. "cardiac arrest" & "laryngoscopy" and I would suggest this paper is of interest to practitioners in anaesthesia / anaesthesiology and intensive care / critical care in addition to emergency medicine. | The keyword list was supplemented according to this suggestion. |
| The abstract is easy to understand and contains the key information from the study. My only reservation is with the assertion that intubation is the "gold standard". There is some work demonstrating that laryngeal mask airways are not inferior for airway management during cardiopulmonary resuscitation with ongoing chest compressions. Your target audience will be aware of this and might prefer a more measured statement e.g. "some patients require intubation as part of their cardiopulmonary resuscitation" or similar. I do note that you have covered this in your introduction with references to support endotracheal intubation but I think it would be reasonable to acknowledge that there is still controversy. This is an opinion rather than a barrier to publication. | Thank you for this hint. Your statement is of course absolutely correct, we have adapted the corresponding statement in the script and refer here to the patient group in which a definitive airway security is sought. |
| The ethics approval is described and it is confirmed that written consent was obtained from participants (which seems appropriate). Unclear what information was given to potential participants and which potential issues were anticipated e.g. what would happen if a participant was concerned after a "poor performance". | We used a hospital-internal standard form for corresponding studies. After the purpose of the study was explained to the participants, they were asked to consent to the use of the video recordings and the data. In addition, the confidentiality of the recordings was assured and the voluntary nature of participation was emphasized. The subjects were made aware that the participation has no influence on the course result and will therefore not have any professional effects. |
| The introduction is short but does explain the background to the study. There are appropriate references. However, there are studies on this topic (VL vs DL during CPR) in real life resuscitation scenarios (e.g. Lee et al., 2015; Park et al., 2015) which have not been referenced. Other studies have only been referred to very briefly. For example, the study by Risse et al. (2020) is referenced but addresses a more similar question than is implied. These studies have typically been observational in nature and not randomised. There are also some randomised studies comparing DL to VL for intubation during CPR (Kim et al., 2016). In addition there are other mannikin / simulation studies addressing this question, including Keresztes et al. who published in this journal in November 2021. It would help if the authors explained how their study relates to these studies. Why have you chosen your methodology to answer the question? What does this study add? Does it tell us something new or does it reinforce the findings of these studies in a different setting or with different equipment or participants? Also, somewhere in the introduction you could clarify that the population considered are adult patients only. | Your references to the relevant literature are very valuable. We emphasized the uniqueness of our study more clearly in the introductory and the discussion part: what makes this study special is the randomized design and the fact that the intubation had to be carried out with ongoing chest compression, which meant that the no-flow time was zero in every case. For this reason, we also chose the FPS as the primary endpoint. |
| The materials and methods section is the bulk of the report and is detailed. I think it would be possible to replicate this study reasonably closely with the information provided. We wondered about a few details e.g. Is there any reason for the number of anaesthetists and anaesthetic nurses included? How did the investigators decide on this? Why were these practitioners chosen (as opposed to say intensivists or paramedics)? Was there a standardised script used for the participants? Were participants blinded to the primary and secondary endpoints? Presumably the answer to the first 3 questions is that it was a pragmatic methodology but it might be reasonable to say this. | The size of the population was fixed for organizational reasons: the participants were employees of our hospital at their annual continuing education course. We designed the study as a "pragmatic" pilot study, which is why we deliberately avoided a power analysis.  Of course, a standardized script was used and the subjects were blinded to the endpoints: they were only told that the study wanted to investigate whether endotracheal intubation could be performed "quickly and safely" with ongoing resuscitation.  We now mention this more clearly in the manuscript. |
| The results section is short but conveys the important results. There are no tables or charts in the body of the report. The average time to intubation and the number of attempts required when there was more than one attempt at intubation are not included. It may be useful to demonstrate the range of intubation attempts in the DL group (especially given 32% in the DL group had to attempt reintubation). | The inclusion of the 9 unsuccessful intubations would increase the difference in the TTI significantly, but this does not correspond to clinical practice: in the case of unsuccessful intubation, interim ventilation using a mask should be given promptly and the next intubation attempt should be made after optimizing the conditions (e.g. positioning the patient, suction) or with an alternative airway device (e.g. SGA). It would therefore be wrong to include these intermediate steps in the pure TTI. |
| The discussion is also short and could include more detail on why first pass intubation and rapid intubation during cardiopulmonary resuscitation are of benefit. It would be reasonable to explain the benefits to patients and practitioners in the range of settings in which patients in cardiac arrest are encountered. You have addressed some limitations to the study but what about downsides to universal videolaryngoscopy? You may not feel there are any significant barriers but this should be explained as readers may not agree. Examples might be cost (particularly in resource limited settings) or portability or maintenance. I note that the methods sections describes the CMAC videolaryngoscope with the screen turned away for the direct laryngoscopy attempts - this indicates that it was not a portable videolaryngoscope used in the study. I don't think this detracts from the findings but could be considered in the discussion. | We've revised this section to better emphasize the benefits of rapid intubation. We also took your valuable advice about the "disadvantages" of the VL seriously and revised the passage here as well. We were also able to justify the use of the external monitor more clearly. |
| The conclusions are reasonable (although they might feel more honest without the intensifiers e.g "support" rather than "strongly support"). | Thank you for this comment. We have reworded the relevant passage. |
| We had a few questions about the tables. These are listed below: Table 1- what does SMD stand for. Table 2 - line 182 includes a spelling mistake - Fist should be FIRST. Table 3 - can "seconds" be changed to "median time in seconds" (for clarity and easy reading). | Thank you very much for these questions. SMD stands for standardized mean difference. We have added this to the list of abbreviations.  Additionally, we have corrected the other two notes in the tables. |
| **Reviewer #3** |  |
| 1. I agree that VL have been shown to provide better glottic view, but not necessarily higher intubation success as well as fastest intubation compared to DL in all cases. It does help in patients with difficult intubations, but not in cases not having any difficulty. Moreover, the success as well as first pass rate varies with different types of DLs. You have not touched this area. Recent meta-analysis on DL including Cochrane one does not echo your whole positive statement on VL compared to DL. | Thank you for pointing this out. As you correctly describe, the better view of the glottis has already been established several times, especially in difficult airways. The extent to which this leads to a better FPS also depends on other circumstances. In our study, however, we were able to show that a significantly higher FPS could be achieved under these specific conditions (simple airway, continued chest compressions).  So that a correct comparison between VL and DL can be investigated in an experiment that is as standardized as possible, we decided to have all subjects intubated with a C-Mac with a “Macintosh”- Blade and to allow the view of the monitor as the only difference. It was not our intention to compare different DL devices.  We have now mentioned these circumstances in more detail in the discussion section. |
| 2. Why participants’ experience so varied? There should have been some standardization. Did you do power analysis as well as subgroup analysis on different level of experiences and categories of those did intubation or not? | The size of the population was given for organizational reasons: the participants were employees of our clinic at their annual continuing education course. This also explains the different experience levels of the participants. However, in Table 1 we were able to show that the groups were statistically comparable (in terms of experience) with an SMD of 0.41. We carried out a stratified randomization between the occupational groups, which is also reflected in the corresponding SMD. As you can see in Table 4, we performed a multivariable logistic regression analysis, which showed no influence of experience or occupation on the primary endpoint. We designed the study as a "pragmatic" pilot study, which is why we deliberately avoided a power analysis. |
| 3. English language used here needs some minor correction as well as editing. Mixture of both UK and USA version of English has been used here. | Thanks for pointing this out, errors have been corrected. |
| 4. I am bit confused about blinding. Though I can understand the randomization, but to my knowledge blinding is difficult between VL and DL. | The study was not a blinded study, but subjects were blinded to the endpoints. Subjects were only told that the study wanted to investigate whether endotracheal intubation could be performed "quickly and safely" with ongoing resuscitation. We now mention this more clearly in the script. |
| 5. How did you blind the assessors? | The assessors were not blinded. However, the video recordings and the internal software of the manikin were used to assess no-flow time objectively. |
| 6. How did you make sure ventilation was effective? Bag ventilation as a method to confirm tracheal intubation is very poor confirmatory test in vivo. | This is an important objection. By using a manikin, we were able to unequivocally demonstrate the position of the tracheal tube with the help of internal detection of ventilation. An oesophageal malposition led to ventilation of the stomach and consequently did not lead to an electronic display of adequate ventilation. We have clarified the corresponding passage in the text. |
| 7. Don’t repeat data of methodology in the result section. | Thank you for this comment. We have reworded the relevant passage. |
| 8. Can you tell me how you have distributed or rather randomised the 28 anaesthetist and nurses? Also, how did you spread their years of experience in two groups? | We performed stratified randomization between physicians and nurses. The experience of the participants did not play into this randomization. However, the analysis of the subjects (Table 1) showed an even distribution between the two groups with an SMD of 0.41. |
| 9. I can see FPS was 100% in VL which is not even found in human trials, neither in published mannequin trial. I am surprised. This raises suspicion on randomization, blinding and assessment methods. I shall like to know whether you have used normal airway or simulated a difficult airway scenario here? | The study was carried out using a patient manikin (Resusci Anne Advanced SkillTrainer, Laerdal Medical, Norway). The same simulators are regularly used at our clinic for training in cardiopulmonary resuscitation. There was no additional artificial aggravation of the airway anatomy.  Macke et al. (doi:10.3390/jcm9092719) showed two years ago in 152 prehospital intubations an FPS of 95% in the VL group and 79% in the DL group and also a difference in the TTI of 3 seconds in favor of the VL. So, an FPS of 100% in a mannikin study doesn't seem unrealistic. |
| 10. The TTI in DL has been proved beyond doubt to be much less than VL, which is opposite here. Can you explain the discrepancy? | In this point we have to disagree with the reviewer. The time to tracheal intubation has not been not been proven to be shorter with direct laryngoscopy compared to video laryngoscopy. A current Cochrane analysis on the use of VL compared to DL for adults undergoing tracheal intubation states that “Thirty-five studies with 4061 participants reported data on time required for tracheal intubation for Macintosh-style VL […] When we combined these studies, we noted considerable statistical heterogeneity (I ^2^ = 96%), which could likely be explained by the variation in the definitions of time for intubation used in the included studies. […] We assessed this outcome to be of very low certainty.” [Hansel J et al. Cochrane Database of Systematic Reviews 2022, Issue 4. Art. No.: CD011136. DOI: 10.1002/14651858.CD011136.pub3.] So, there is still considerable uncertainty whether VL or DL requires less time to tracheal intubation. |
| 11. Discussion should not start with aim which should be mentioned in the introductory section, rather discussion should start with most important positive and negative findings. | We have deleted the relevant sentence and adapted the wording of the discussion. |
| 12. ETI is not recommended as first line of important part of management in CPR unless the oxygenation or ventilation is an issue. Airway can even be managed with supraglottic devices in case airway control is needed. ETI or advanced airway is only recommended after 2-3 cycles, if at all necessary, as per current ACLS protocol. I do not agree with your statement regarding securing airway at the earliest. | Your statement is of course absolutely correct, we have adapted the corresponding statement in the script and refer here to the patient group in which a definitive airway security is sought.  Please see also our comments above regarding this issue (answers to Reviewer 1 and 2). |
| 13. VL is not easily available and readily set up in emergency situation, rather it has utility in elective difficult airway scenario. Setting a VL takes time and manpower which is not practical in most cases. Also, the efficacy in struggling CPR time in real life scenario is not proven with VL, where DL is still gold-standard. I refuse to accept your concluding statement here. | On this point we have to respectfully disagree. Most of the authors have well-founded experience in the preclinical setting: if the material is arranged appropriately in the emergency bag, the preparation for intubation with a video laryngoscope (especially a monitor-on-handle device) takes no longer than for conventional intubation.  For example, Hossfeld et al. in 2020 (doi: 10.1097/EJA.0000000000001286) describe that the VL C-Mac is used in German HEMS as the first-line device. Likewise, Knapp et al. (doi:10.1186/s13049-021-00863-9) note that intubation at the Swiss Air Rescue Service (REGA) must be carried out using a VL according the standard operating procedures. |
| 14. Success of securing airway in critically ill lifesaving urgent set ups should not be assessed using beginners or newcomers in airway management. This is poor planning. | We agree that, in the clinical setting, airway management in critically ill and unstable patients must be performed by the most experienced practitioner. However, the participants of our study were all anaesthetists and anaesthesia nurses and had a working experience of a median of 5 years (doctors)/15 years (nurses) in anaesthesia, with a minimum of 0.75 years (doctor)/2 years (nurses) with an estimated average minimum of 1 tracheal intubations per working day. So, compared to most prehospital critical care providers and staff in the emergency department, the participants of our study were very well trained in tracheal intubation.  Even in this relatively well-trained group of providers, we were able to prove the benefit of the use of VL. |
| 15. Many other limitations not mentioned including flaws in design, conduct, as well as data analysis. | As mentioned earlier, we have now made the limitations of the study more explicit in the discussion.  Thank you very much for the critical review of our manuscript. |
